# 3-Methyl-1-(3-nitro­phen­yl)-5-phenyl-4,5-dihydro-1*H*-pyrazole

**DOI:** 10.1107/S1600536809031390

**Published:** 2009-08-15

**Authors:** Jun-qiang Chen, He-ping Li, Chang-shan Huang, Jin-ying Wu

**Affiliations:** a Energy Research Institute Co Ltd, Henan Academy of Sciences, Zhengzhou 450000, People’s Republic of China; b School of Chemistry and Biological Engineering, Guilin University of Technology, People’s Republic of China

## Abstract

In the title compound, C_16_H_15_N_3_O_2_, the planar [maximum deviation 0.156 (2) Å] pyrazoline ring is nearly coplanar with the 3-nitro­phenyl group and is approximately perpendicular to the phenyl ring, making dihedral angles of 3.80 (8) and 80.58 (10)°, respectively. Weak inter­molecular C—H⋯O hydrogen bonding is present in the crystal structure.

## Related literature

For applications of pyrazoline derivatives, see: Hatheway *et al.* (1978 ▶); Mahajan *et al.* (1991 ▶); Sobczak & Pawlaczyk (1998 ▶).
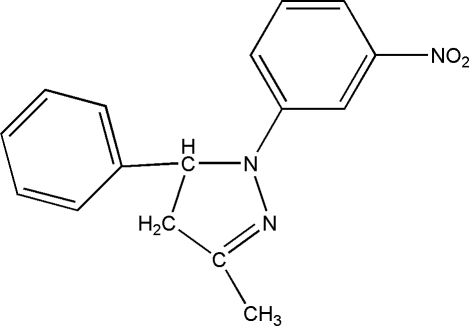


## Experimental

### 

#### Crystal data

C_16_H_15_N_3_O_2_
*M* *_r_* = 281.31
Monoclinic, 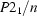

*a* = 12.0173 (4) Å
*b* = 7.9324 (2) Å
*c* = 15.4944 (5) Å
β = 99.160 (2)°
*V* = 1458.18 (8) Å^3^
*Z* = 4
Mo *K*α radiation
μ = 0.09 mm^−1^
*T* = 296 K
0.36 × 0.18 × 0.07 mm

#### Data collection

Bruker SMART CCD area-detector diffractometer
Absorption correction: none
10272 measured reflections
3014 independent reflections
1648 reflections with *I* > 2σ(*I*)
*R*_int_ = 0.034

#### Refinement

*R*[*F* ^2^ > 2σ(*F* ^2^)] = 0.047
*wR*(*F* ^2^) = 0.128
*S* = 1.00
3014 reflections
190 parameters
H-atom parameters constrained
Δρ_max_ = 0.14 e Å^−3^
Δρ_min_ = −0.20 e Å^−3^

### 

Data collection: *SMART* (Bruker, 1998 ▶); cell refinement: *SAINT* (Bruker, 1998 ▶); data reduction: *SAINT* (Bruker, 1998 ▶); program(s) used to solve structure: *SHELXS97* (Sheldrick, 2008 ▶); program(s) used to refine structure: *SHELXL97* (Sheldrick, 2008 ▶); molecular graphics: *ORTEP-3 for Windows* (Farrugia, 1997 ▶); software used to prepare material for publication: *WinGX* (Farrugia, 1999 ▶).

## Supplementary Material

Crystal structure: contains datablocks global, I. DOI: 10.1107/S1600536809031390/xu2579sup1.cif

Structure factors: contains datablocks I. DOI: 10.1107/S1600536809031390/xu2579Isup2.hkl

Additional supplementary materials:  crystallographic information; 3D view; checkCIF report

## Figures and Tables

**Table 1:** Hydrogen-bond geometry (Å, °)

*D*—H⋯*A*	*D*—H	H⋯*A*	*D*⋯*A*	*D*—H⋯*A*
C14—H14*A*⋯O1^i^	0.93	2.51	3.245 (2)	136

Symmetry code: (i) 
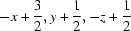
.

**Fig. 1.**
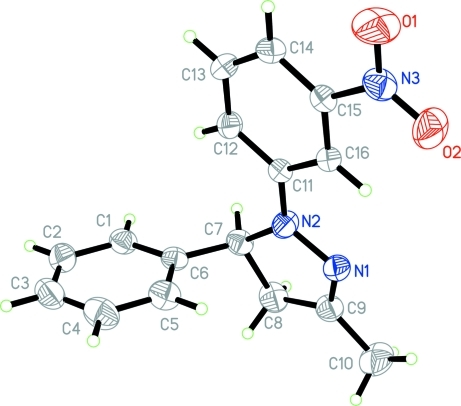
The molecular structure of the compound. The displacement ellipsoids are drawn at the 30% probability level.

**Fig. 2.**
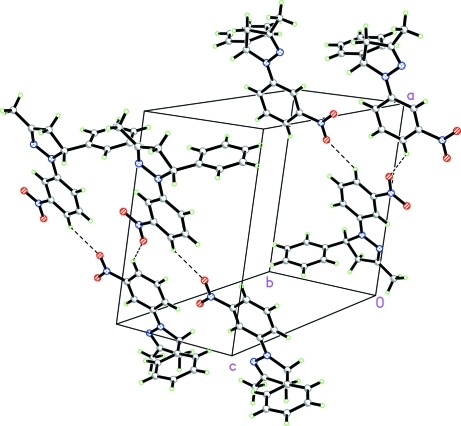
Packing of (I), showing the intermolecular hydrogen bonds as dashed lines.

## supplementary crystallographic information

### Comment

The derivatives of pyrazoline are mostly used in medicine, for example as antitumor (Hatheway *et al.*, 1978), analgesic (Sobczak & Pawlaczyk, 1998), and antimicrobial (Mahajan *et al.*, 1991) agents. As part of our work, the title compound is recently synthesized in our group and its crystal structure is reported here.

The pyrazoline ring and the 3-nitrophenyl ring are nearly coplanar, making a dihedral angle of 3.80 (8)°, while the dihedral angle between the pyrazoline ring and the C1-phenyl ring is 80.58 (10)° (Fig. 1). Intermolecular weak C—H···O hydrogen bonding is present in the crystal structure (Fig. 2 and Table 1).

### Experimental

3-Nitrophenylhydrazine (1 mmol, 0.153 g) was dissolved in anhydrous ethanol (15 ml). The mixture was stirred for several min at 351 K, benzylideneacetone (1 mmol, 0.146 g) in ethanol (8 ml) was added dropwise and the mixture was stirred at refluxing temperature for 2 h. The product was isolated and recrystallized from methanol, red single crystals were obtained after 2 d.

### Refinement

All H atoms were positioned geometrically and refined as riding with C—H = 0.93 (aromatic), 0.97 (methylene), 0.98 (methine) and 0.96 Å (methyl), with *U*_iso_(H) = 1.5*U*_eq_(C) for methyl H atoms and 1.2*U*_eq_(C) for the others.

### Crystal data

**Table:**

C_16_H_15_N_3_O_2_	*F*(000) = 592
*M**_r_* = 281.31	*D*_x_ = 1.281 Mg m^−^^3^
Monoclinic, *P*2_1_/*n*	Mo *K*α radiation, λ = 0.71073 Å
Hall symbol: -P 2yn	Cell parameters from 1824 reflections
*a* = 12.0173 (4) Å	θ = 2.6–26.5°
*b* = 7.9324 (2) Å	µ = 0.09 mm^−^^1^
*c* = 15.4944 (5) Å	*T* = 296 K
β = 99.160 (2)°	Plate, red
*V* = 1458.18 (8) Å^3^	0.36 × 0.18 × 0.07 mm
*Z* = 4	

### Data collection

**Table:**

Bruker SMART CCD area-detector diffractometer	1648 reflections with *I* > 2σ(*I*)
Radiation source: fine-focus sealed tube	*R*_int_ = 0.034
graphite	θ_max_ = 26.5°, θ_min_ = 2.0°
ω scans	*h* = −14→15
10272 measured reflections	*k* = −9→8
3014 independent reflections	*l* = −18→19

### Refinement

**Table:**

Refinement on *F*^2^	Primary atom site location: structure-invariant direct methods
Least-squares matrix: full	Secondary atom site location: difference Fourier map
*R*[*F*^2^ > 2σ(*F*^2^)] = 0.047	Hydrogen site location: inferred from neighbouring sites
*wR*(*F*^2^) = 0.128	H-atom parameters constrained
*S* = 1.00	*w* = 1/[σ^2^(*F*_o_^2^) + (0.0621*P*)^2^] where *P* = (*F*_o_^2^ + 2*F*_c_^2^)/3
3014 reflections	(Δ/σ)_max_ < 0.001
190 parameters	Δρ_max_ = 0.14 e Å^−^^3^
0 restraints	Δρ_min_ = −0.20 e Å^−^^3^

### Special details

**Table:**

Geometry. All e.s.d.'s (except the e.s.d. in the dihedral angle between two l.s. planes) are estimated using the full covariance matrix. The cell e.s.d.'s are taken into account individually in the estimation of e.s.d.'s in distances, angles and torsion angles; correlations between e.s.d.'s in cell parameters are only used when they are defined by crystal symmetry. An approximate (isotropic) treatment of cell e.s.d.'s is used for estimating e.s.d.'s involving l.s. planes.
Refinement. Refinement of *F*^2^ against ALL reflections. The weighted *R*-factor *wR* and goodness of fit *S* are based on *F*^2^, conventional *R*-factors *R* are based on *F*, with *F* set to zero for negative *F*^2^. The threshold expression of *F*^2^ > σ(*F*^2^) is used only for calculating *R*-factors(gt) *etc*. and is not relevant to the choice of reflections for refinement. *R*-factors based on *F*^2^ are statistically about twice as large as those based on *F*, and *R*- factors based on ALL data will be even larger.

### Fractional atomic coordinates and isotropic or equivalent isotropic displacement parameters (Å^2^)

**Table:**

	*x*	*y*	*z*	*U*_iso_*/*U*_eq_	
C11	0.40009 (13)	0.17187 (19)	0.06087 (10)	0.0433 (4)	
N2	0.29773 (12)	0.18897 (16)	0.00749 (10)	0.0578 (4)	
C15	0.54447 (14)	0.00161 (19)	0.13960 (10)	0.0454 (4)	
C7	0.24060 (14)	0.3481 (2)	−0.02008 (11)	0.0535 (5)	
H7A	0.2879	0.4156	−0.0529	0.064*	
C16	0.44095 (13)	0.01314 (19)	0.08743 (10)	0.0425 (4)	
H16A	0.3991	−0.0832	0.0703	0.051*	
N1	0.23515 (12)	0.04742 (18)	−0.02230 (9)	0.0548 (4)	
C12	0.46581 (14)	0.3125 (2)	0.08824 (11)	0.0519 (5)	
H4A	0.4395	0.4197	0.0714	0.062*	
C6	0.21138 (13)	0.4493 (2)	0.05545 (11)	0.0472 (4)	
C13	0.56934 (14)	0.2941 (2)	0.13993 (12)	0.0561 (5)	
H13A	0.6121	0.3895	0.1572	0.067*	
N3	0.58488 (14)	−0.1672 (2)	0.16800 (11)	0.0619 (4)	
C1	0.21969 (14)	0.6221 (2)	0.05691 (13)	0.0584 (5)	
H1B	0.2456	0.6777	0.0111	0.070*	
O1	0.67543 (13)	−0.17917 (18)	0.21576 (11)	0.0990 (6)	
C14	0.61085 (14)	0.1383 (2)	0.16652 (11)	0.0536 (5)	
H14A	0.6809	0.1259	0.2013	0.064*	
O2	0.52813 (13)	−0.28871 (17)	0.14279 (11)	0.0905 (5)	
C5	0.17261 (15)	0.3710 (3)	0.12393 (13)	0.0650 (5)	
H5A	0.1664	0.2542	0.1241	0.078*	
C8	0.13676 (16)	0.2830 (2)	−0.08217 (13)	0.0684 (6)	
H8A	0.0675	0.3240	−0.0649	0.082*	
H8B	0.1391	0.3168	−0.1420	0.082*	
C9	0.14648 (15)	0.0972 (2)	−0.07219 (12)	0.0591 (5)	
C4	0.14288 (17)	0.4619 (3)	0.19199 (14)	0.0777 (6)	
H12A	0.1166	0.4065	0.2377	0.093*	
C2	0.19016 (16)	0.7141 (3)	0.12524 (16)	0.0729 (6)	
H2A	0.1963	0.8310	0.1255	0.088*	
C3	0.15175 (17)	0.6329 (4)	0.19279 (14)	0.0777 (6)	
H3A	0.1318	0.6945	0.2391	0.093*	
C10	0.06227 (18)	−0.0247 (3)	−0.11675 (16)	0.0957 (8)	
H10A	0.0856	−0.1375	−0.1002	0.144*	
H10B	0.0567	−0.0124	−0.1789	0.144*	
H10C	−0.0098	−0.0029	−0.0999	0.144*	

### Atomic displacement parameters (Å^2^)

**Table:**

	*U*^11^	*U*^22^	*U*^33^	*U*^12^	*U*^13^	*U*^23^
C11	0.0398 (10)	0.0436 (10)	0.0470 (10)	0.0024 (8)	0.0079 (8)	−0.0019 (8)
N2	0.0495 (9)	0.0418 (8)	0.0756 (11)	0.0045 (7)	−0.0098 (8)	−0.0003 (7)
C15	0.0460 (10)	0.0452 (10)	0.0457 (10)	0.0054 (8)	0.0097 (8)	0.0021 (8)
C7	0.0505 (11)	0.0526 (11)	0.0567 (11)	0.0095 (9)	0.0057 (9)	0.0079 (9)
C16	0.0402 (10)	0.0422 (10)	0.0451 (10)	−0.0005 (7)	0.0066 (8)	−0.0016 (7)
N1	0.0486 (9)	0.0553 (9)	0.0586 (10)	−0.0003 (7)	0.0028 (8)	−0.0044 (7)
C12	0.0524 (11)	0.0414 (10)	0.0613 (12)	0.0019 (8)	0.0072 (9)	−0.0004 (8)
C6	0.0393 (10)	0.0503 (11)	0.0509 (11)	0.0056 (8)	0.0044 (8)	0.0076 (8)
C13	0.0483 (11)	0.0520 (11)	0.0669 (13)	−0.0111 (9)	0.0059 (10)	−0.0090 (9)
N3	0.0575 (10)	0.0596 (11)	0.0673 (11)	0.0122 (9)	0.0054 (9)	0.0115 (9)
C1	0.0515 (11)	0.0548 (12)	0.0698 (13)	0.0035 (9)	0.0127 (10)	0.0041 (10)
O1	0.0680 (10)	0.0945 (12)	0.1213 (13)	0.0163 (8)	−0.0254 (10)	0.0320 (9)
C14	0.0414 (10)	0.0600 (12)	0.0576 (12)	0.0024 (9)	0.0020 (9)	−0.0024 (9)
O2	0.0924 (11)	0.0471 (8)	0.1237 (14)	0.0038 (8)	−0.0081 (10)	0.0076 (8)
C5	0.0650 (13)	0.0650 (12)	0.0661 (13)	0.0024 (10)	0.0139 (11)	0.0113 (11)
C8	0.0642 (13)	0.0779 (14)	0.0579 (13)	0.0157 (11)	−0.0064 (10)	−0.0013 (10)
C9	0.0494 (11)	0.0694 (13)	0.0555 (12)	0.0051 (10)	−0.0012 (10)	−0.0062 (10)
C4	0.0732 (15)	0.1004 (19)	0.0631 (15)	0.0073 (13)	0.0215 (12)	0.0091 (13)
C2	0.0633 (13)	0.0636 (13)	0.0913 (17)	0.0054 (11)	0.0102 (13)	−0.0148 (12)
C3	0.0609 (13)	0.1077 (19)	0.0644 (15)	0.0120 (13)	0.0094 (11)	−0.0195 (14)
C10	0.0690 (15)	0.1000 (18)	0.1050 (19)	−0.0036 (12)	−0.0269 (13)	−0.0190 (14)

### Geometric parameters (Å, °)

**Table:**

C11—N2	1.375 (2)	N3—O2	1.2093 (18)
C11—C16	1.390 (2)	N3—O1	1.2185 (19)
C11—C12	1.393 (2)	C1—C2	1.378 (3)
N2—N1	1.3888 (18)	C1—H1B	0.9300
N2—C7	1.4678 (19)	C14—H14A	0.9300
C15—C14	1.371 (2)	C5—C4	1.371 (3)
C15—C16	1.374 (2)	C5—H5A	0.9300
C15—N3	1.468 (2)	C8—C9	1.485 (3)
C7—C6	1.506 (2)	C8—H8A	0.9700
C7—C8	1.539 (2)	C8—H8B	0.9700
C7—H7A	0.9800	C9—C10	1.488 (3)
C16—H16A	0.9300	C4—C3	1.360 (3)
N1—C9	1.275 (2)	C4—H12A	0.9300
C12—C13	1.376 (2)	C2—C3	1.370 (3)
C12—H4A	0.9300	C2—H2A	0.9300
C6—C5	1.373 (2)	C3—H3A	0.9300
C6—C1	1.374 (2)	C10—H10A	0.9600
C13—C14	1.371 (2)	C10—H10B	0.9600
C13—H13A	0.9300	C10—H10C	0.9600
			
N2—C11—C16	120.49 (14)	C6—C1—H1B	119.5
N2—C11—C12	120.89 (14)	C2—C1—H1B	119.5
C16—C11—C12	118.61 (15)	C15—C14—C13	117.08 (16)
C11—N2—N1	120.35 (13)	C15—C14—H14A	121.5
C11—N2—C7	126.29 (14)	C13—C14—H14A	121.5
N1—N2—C7	113.28 (13)	C4—C5—C6	121.3 (2)
C14—C15—C16	123.67 (15)	C4—C5—H5A	119.4
C14—C15—N3	118.79 (15)	C6—C5—H5A	119.4
C16—C15—N3	117.54 (15)	C9—C8—C7	103.05 (14)
N2—C7—C6	112.85 (14)	C9—C8—H8A	111.2
N2—C7—C8	100.85 (13)	C7—C8—H8A	111.2
C6—C7—C8	113.49 (13)	C9—C8—H8B	111.2
N2—C7—H7A	109.8	C7—C8—H8B	111.2
C6—C7—H7A	109.8	H8A—C8—H8B	109.1
C8—C7—H7A	109.8	N1—C9—C8	114.49 (16)
C15—C16—C11	118.59 (15)	N1—C9—C10	121.42 (18)
C15—C16—H16A	120.7	C8—C9—C10	124.09 (17)
C11—C16—H16A	120.7	C3—C4—C5	120.1 (2)
C9—N1—N2	107.90 (15)	C3—C4—H12A	120.0
C13—C12—C11	120.57 (15)	C5—C4—H12A	120.0
C13—C12—H4A	119.7	C3—C2—C1	119.8 (2)
C11—C12—H4A	119.7	C3—C2—H2A	120.1
C5—C6—C1	118.09 (17)	C1—C2—H2A	120.1
C5—C6—C7	120.62 (16)	C4—C3—C2	119.8 (2)
C1—C6—C7	121.27 (16)	C4—C3—H3A	120.1
C14—C13—C12	121.49 (16)	C2—C3—H3A	120.1
C14—C13—H13A	119.3	C9—C10—H10A	109.5
C12—C13—H13A	119.3	C9—C10—H10B	109.5
O2—N3—O1	122.49 (16)	H10A—C10—H10B	109.5
O2—N3—C15	119.20 (15)	C9—C10—H10C	109.5
O1—N3—C15	118.31 (16)	H10A—C10—H10C	109.5
C6—C1—C2	120.93 (19)	H10B—C10—H10C	109.5

### Hydrogen-bond geometry (Å, °)

**Table:**

*D*—H···*A*	*D*—H	H···*A*	*D*···*A*	*D*—H···*A*
C14—H14A···O1^i^	0.93	2.51	3.245 (2)	136

Symmetry codes: (i) −*x*+3/2, *y*+1/2, −*z*+1/2.
